# Immune profiling reveals prognostic genes in high-grade serous ovarian cancer

**DOI:** 10.18632/aging.103199

**Published:** 2020-06-16

**Authors:** Yong Wu, Lingfang Xia, Ping Zhao, Yu Deng, Qinhao Guo, Jun Zhu, Xiaojun Chen, Xingzhu Ju, Xiaohua Wu

**Affiliations:** 1 Department of Gynecologic Oncology, Fudan University Shanghai Cancer Center, Shanghai, China; 2 Department of Oncology, Shanghai Medical College, Fudan University, Shanghai, China; 3 Department of Pathology, Ruijin Hospital, Shanghai Jiaotong University School of Medicine, Shanghai, China; 4 Department of Pathology, The First Affiliated Hospital, Zhejiang University School of Medicine, Hangzhou, China

**Keywords:** high-grade serous ovarian cancer, tumor microenvironment score, TCGA, ICGC, overall survival

## Abstract

High-grade serous ovarian cancer (HGSOC) is a heterogeneous disease with diverse clinical outcomes, highlighting a need for prognostic biomarker identification. Here, we combined tumor microenvironment (TME) scores with HGSOC characteristics to identify immune-related prognostic genes through analysis of gene expression profiles and clinical patient data from The Cancer Genome Atlas and the International Cancer Genome Consortium public cohorts. We found that high TME scores (TMEscores) based on the fractions of immune cell types correlated with better overall survival. Furthermore, differential expression analysis revealed 329 differentially expressed genes between patients with high vs. low TMEscores. Gene Ontology and Kyoto Encyclopedia of Genes and Genomes analyses showed that these genes participated mainly in immune-related functions and, among them, 48 TME-related genes predicted overall survival in HGSOC. Seven of those genes were associated with prognosis in an independent HGSOC database. Finally, the two genes with the lowest *p*-values in the prognostic analysis (GBP1, ETV7) were verified through *in vitro* experiments. These findings reveal specific TME-related genes that could serve as effective prognostic biomarkers for HGSOC.

## INTRODUCTION

Among gynecologic malignancies, ovarian cancer (OC) has a poor prognosis. High-grade serous ovarian cancer (HGSOC), the most common epithelial OC, is usually diagnosed in an advanced stage [1, 2], and patients have widely different outcomes in spite or showing equivalent clinical or pathologic characteristics. Conventional clinical features, such as CA-125 levels, do not accurately predict HGSOC prognosis [3]. Given the genetic heterogeneity of patients, identifying prognostic biomarkers may improve patient outcomes. The availability of large-scale public cohorts offers the opportunity to explore the association between gene expression and clinical prognosis [4–6].

Accumulated evidence indicates that the tumor microenvironment (TME) can help predict survival outcomes and assess therapeutic efficacy [7–10], although genetic and epigenetic changes may contribute to progression and recurrence in different cancer types. The state of the patient’s immune system can be a determining factor of cancer initiation and progression. In recent years, immune profiling has come to the forefront in cancer research [11, 12], with studies based on immunoscores providing prognostic parameters for various types of solid tumors [13]. Therefore, using computer-based analysis to explore the relationship between the TME and prognosis in HGSOC may improve the clinical management of HGSOC.

CIBERSORT is a deconvolution algorithm that uses a set of reference gene expression values (a signature with 547 genes) considered a minimal representation for each cell type and, based on those values, uses a support vector regression method to infer cell type proportions in data from bulk tumor samples containing mixed cell types [14, 15]. Here, for the first time, by taking advantage of both public databases of HGSOC cohorts and CIBERSORT algorithm-derived TMEscore values, we extracted a list of TME-related genes that can predict the survival of HGSOC patients. Furthermore, we employed molecular experiments to confirm the *in vitro* functions and prognostic value of this profile.

## RESULTS

### Study design and patient selection

**Figure 1:**
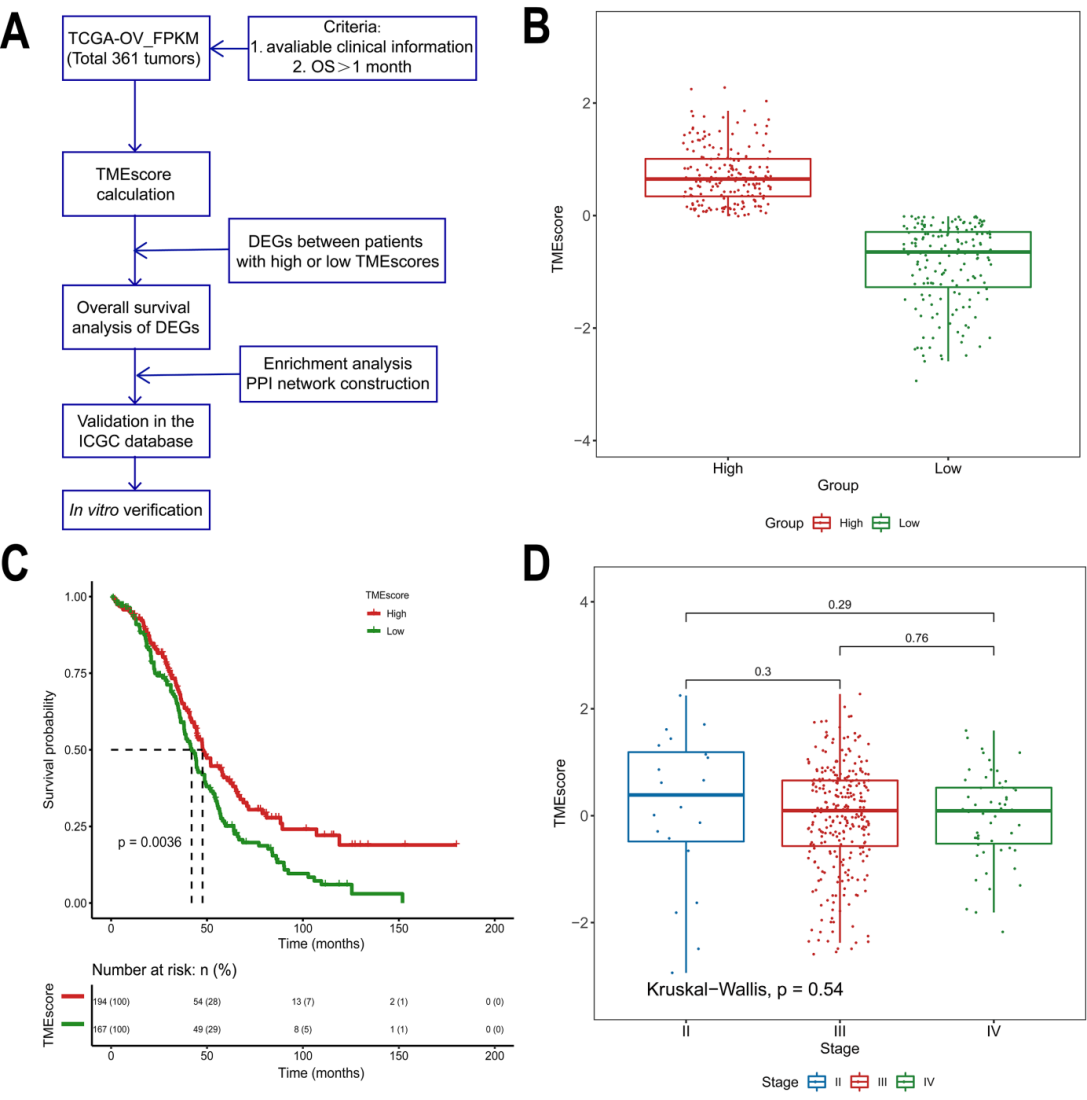
**Association between the TMEscore and prognosis in TCGA data analyzed by the CIBERSORT algorithm.** (**A**) Workflow of the current study. (**B**) Based on the median TMEscore values, patients with HGSOC were divided into the high and low TMEscore groups. (**C**) As shown in the Kaplan-Meier plot, the median survival time in the high TMEscore group was longer than that in the low score group (*p*=0.0036). (**D**) Distribution of TMEscores by tumor stage for HGSOC patients. The boxplot shows that there was no association between tumor stage and TMEscore.

The overall scheme is shown in Figure 1A. A total of 361 ovarian cancer samples were enrolled according to the criteria (eligible clinical information, overall survival > 1 month). Patient characteristics obtained from The Cancer Genome Atlas (TCGA) are detailed in Table 1. In general, we assessed the prognostic value of the TMEscore and then attempted to identify TME-related differentially expressed genes (DEGs) that contribute to HGSOC overall survival in the TCGA database. DEGs were further validated in the International Cancer Genome Consortium (ICGC), a separate HGSOC database.

**Table 1:** Clinicopathological characteristics of 361 patients with HGSOC.

**Parameter**	**Subtype**	**Patients (n)**
Age	>=59	182
	<59	179
Stage	I	1
	II	20
	III	283
	IV	54
	Not available	3
OS time (months)	<12	65
	>=12	296

### TMEscore is associated with overall survival in HGSOC

Of all HGSOC samples, 361 samples were evaluated. As calculated using the CIBERSORT algorithm, the TMEscore values ranged from -2.93883 to 2.27524 (Supplementary Table 1). Based on the median TMEscore values, 194 patients were in the high score group (53.7%), while 167 were in the low score group (46.3%, Figure 1B). The associations between the TMEscore and corres-ponding overall survival were then analyzed by generating Kaplan-Meier plots and evaluating the data with a log-rank test. The Kaplan-Meier plots demonstrated that a high TMEscore was positively correlated with favorable survival in HGSOC patients (Figure 1C, *p*=0.0036). Further evaluation revealed no correlation between TMEscore and tumor stage (*p*=0.54, Figure 1D).

### DEGs between the TMEscore groups

**Figure 2:**
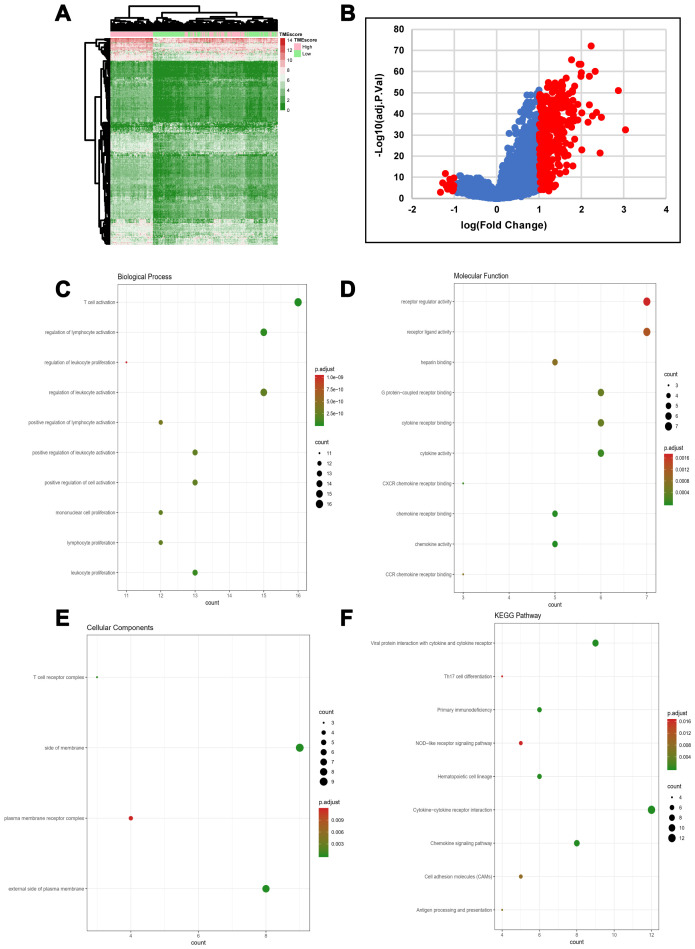
**DEG profiles by TMEscore in HGSOC.** (**A**) Heatmap of the DEGs between the top half (high score) vs. bottom half (low score) of TMEscore values. A |log(fold change)|≥1 and an adjusted p-value < 0.05 were set as the cutoff criteria to screen for DEGs. (**B**) Volcano plot of gene expression profile data for patients with high and low TMEscores. (**C**–**F**) Functional enrichment analysis including Biological Process (BP), Cellular Components (CC), and Molecular Functions (MF) categories as well as KEGG pathways for 329 DEGs.

We computed a heatmap to compare differential gene expression profiles in the high and low TMEscore groups (Figure 2A). We identified a total of 329 DEGs, including 311 upregulated and 18 downregulated genes (Supplementary Table 2). A volcano plot of gene expression profile data between patients with a high or low TMEscore is shown in Figure 2B. We carried out biological enrichment analyses, including Gene ontology (GO) and Kyoto Encyclopedia of Genes and Genomes (KEGG) pathway analyses, to outline the potential functions of DEGs. These genes were mainly involved in immune-related functions (Figure 2C–2F), such as T cell activation, cytokine receptor binding, T cell receptor complex and Th17 cell differentiation, suggesting that DEGs might exert functions in immune-related pathways in HGSOC.

### Survival analysis of DEGs in HGSOC

**Figure 3:**
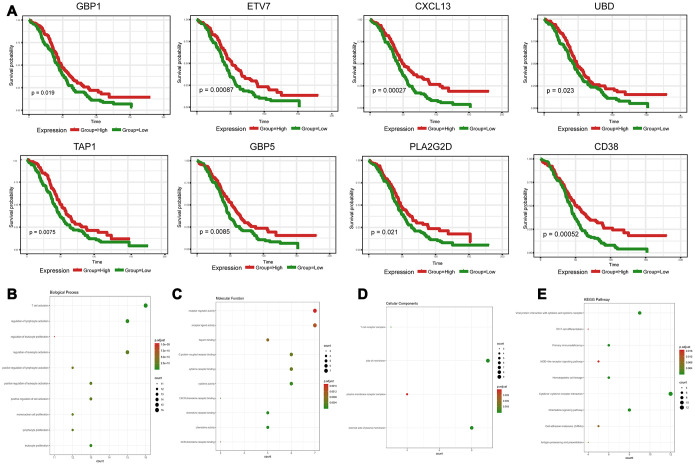
**Discovery of prognostic TME-related DEGs with functional annotations in TCGA.** (**A**) Kaplan-Meier survival curves were generated for selected DEGs with prognostic significance by a log-rank test. OS=overall survival time in months. (**B**–**E**) GO term and KEGG pathway analyses with the 48 prognostic DEGs.

To explore the clinical role of individual DEGs in overall survival, we generated Kaplan-Meier survival curves from TCGA data. Based on the median value of each DEG, a total of 48 genes were shown to predict overall survival (Supplementary Table 3; *p*<0.05). The survival curves for the prognostic genes are shown in Figure 3A and Supplementary Figure 1. Consequently, enrichment analyses were performed with the prognostic DEGs. KEGG and GO analyses indicated that these genes were mainly involved in immune pathways such asTh17 cell differentiation, Cytokine−cytokine receptor interaction and Chemokine signaling pathways, similar to previous enrichment analysis of DEGs (Figure 3B–3E).

### Protein-protein interaction (PPI) network among prognostic genes

**Figure 4:**
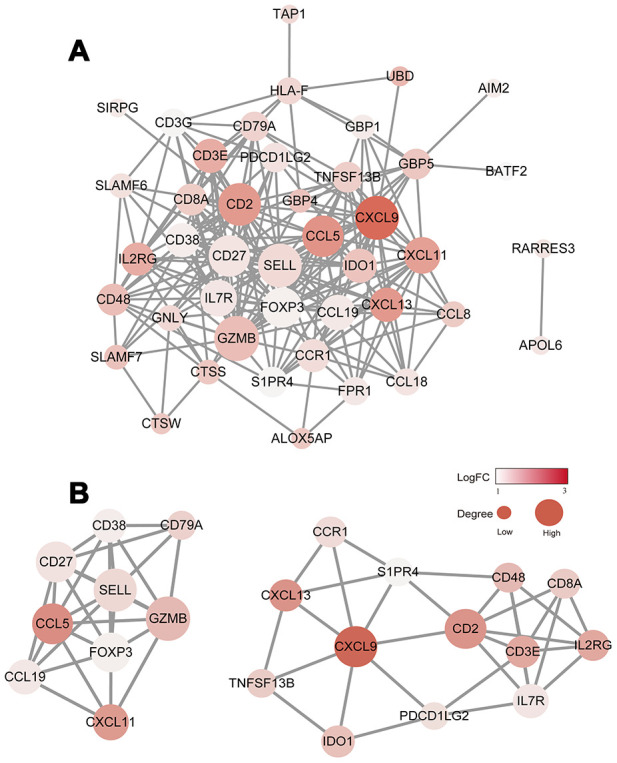
**Construction of the PPI network for the 48 prognostic DEGs.** (**A**) The PPI network was constructed using the 48 prognostic DEGs with the R software package STRINGdb. (**B**) MCODE was used to identify the main coregulated modules. The most significant module is indicated in two closely related subgroups.

To explore the interplay among the 48 DEGs, we used the STRING database (https://string-db.org/) to analyze protein network interactions. We obtained a relationship network containing 222 edges and 43 nodes (Figure 4A). Moreover, in the PPI network, CXCL9, CXCL13, CCL5, GZMB and CD2 were the most remarkable nodes as they had the most connections with other nodes. Molecular COmplex DEtection (MCODE), a Cytoscape plugin, was used to identify the main coregulated modules. By applying the MCODE tool, we identified two closely related subgroups (Figure 4B). As shown in Figure 4B, we can see that all the module genes are up-regulated genes of DEGs, including CXCL9, CCL5, CXCL11, CD27, FOXP3, CD8A and other immune-related genes. The connected nodes for each gene intersection are listed in Supplementary Table 4.

### Prognostic validation in the ICGC database

**Figure 5:**
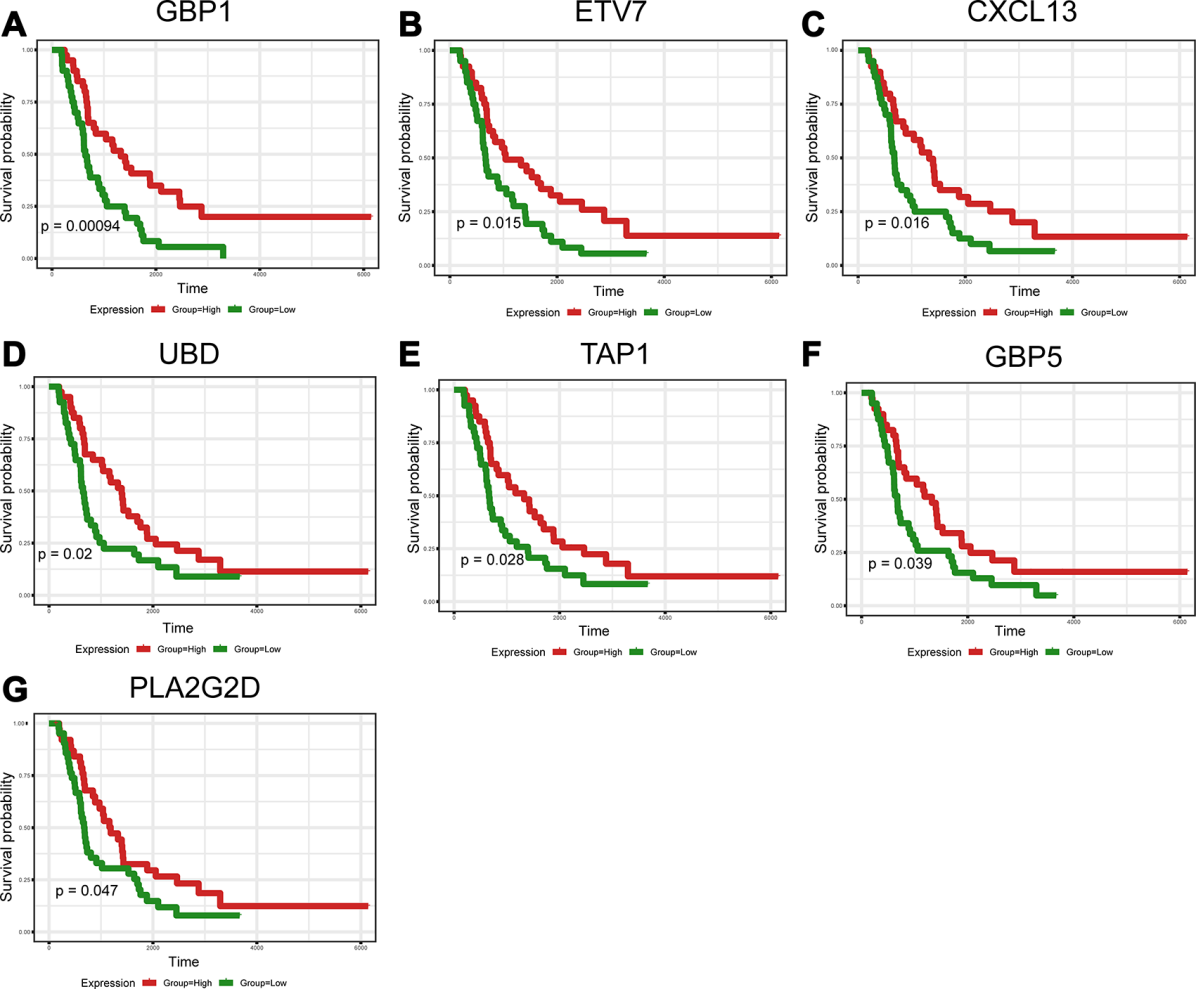
**Validation of prognostic DEGs extracted from the TCGA database in the ICGC cohort.** (**A**–**G**) Kaplan-Meier survival curves were generated for seven validated DEGs in an additional cohort of 81 HGSOC patients from the ICGC. *p*<0.05 by a log-rank test. OS=overall survival time in days.

To further validate the prognostic significance of 48 genes identified from the TCGA database, we downloaded and analyzed the gene expression data of a cohort of 81 HGSOC patients from the ICGC (Supplementary Table 5), an independent HGSOC database. Forty-seven of the 48 genes identified above had expression values, and further analyses validated seven genes (*p*<0.05, Figure 5A–5G) associated with prognosis (Supplementary Table 6). Notably, elevated mRNA levels of these genes correlated with better patient OS.

### GBP1, ETV7 and CXCL13 perform molecular functions *in vitro*

**Figure 6:**
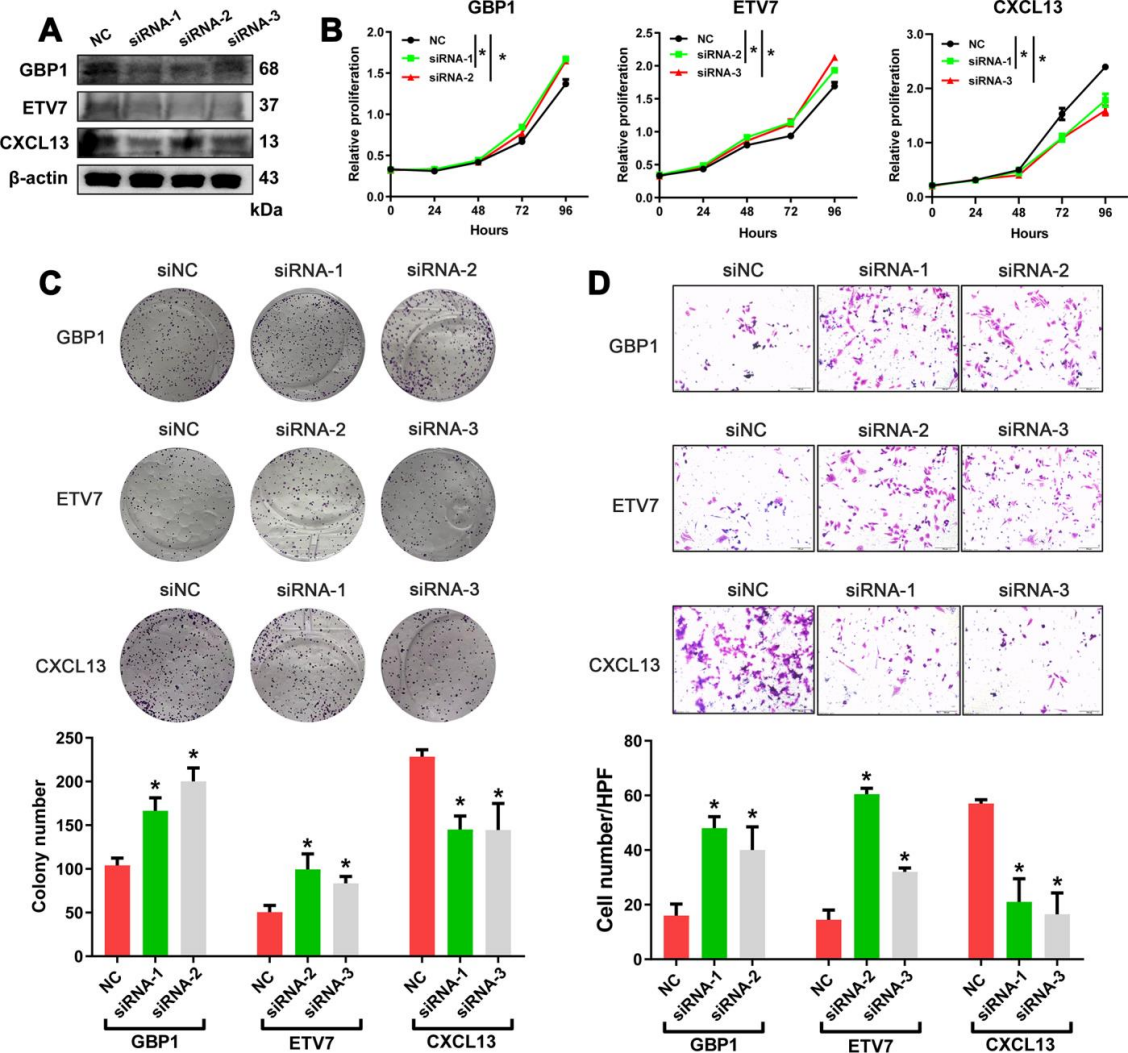
***In vitro* assessment of GBP1, ETV7 and CXCL13.** (**A**) The protein levels of GBP1, ETV7 and CXCL13 were measured by western blotting in A2780 cells transfected with siRNAs. (**B**) CCK-8 assays were performed to evaluate the proliferation of GBP1-, ETV7- and CXCL13-knockdown cells. (**C**) The colony-forming ability of A2780 cells was assessed to determine the effects of GBP1, ETV7 or CXCL13 downregulation on cell growth. (**D**) The invasion potential of cells was assessed using a Transwell assay. The scale bar represents 100 μm. NC: negative control. * indicates *p*< 0.05.

Crosstalk between tumor cells and their surrounding microenvironment is necessary for cell survival, growth, and proliferation and for epithelial-mesenchymal transition (EMT) and metastasis [16–18]. We sought to examine the molecular functions of these genes. The three genes with the lowest *p*-values validated from the ICGC database were selected for functional assays in HGSOC cells. A2780 cells were separately transduced with small interfering RNAs (siRNAs) targeting these three genes, and western blotting confirmed the efficiency of the siRNAs in A2780 cells (Figure 6A). The results showed that knockdown of GBP1 and ETV7 promoted the proliferation, colony formation, and migration of cells (Figure 6B–6D). In contrast, the opposite effects were observed when CXCL13 was downregulated (Figure 6B–6D).

### Confirmation of the expression levels of GBP1, ETV7 in patients

**Figure 7:**
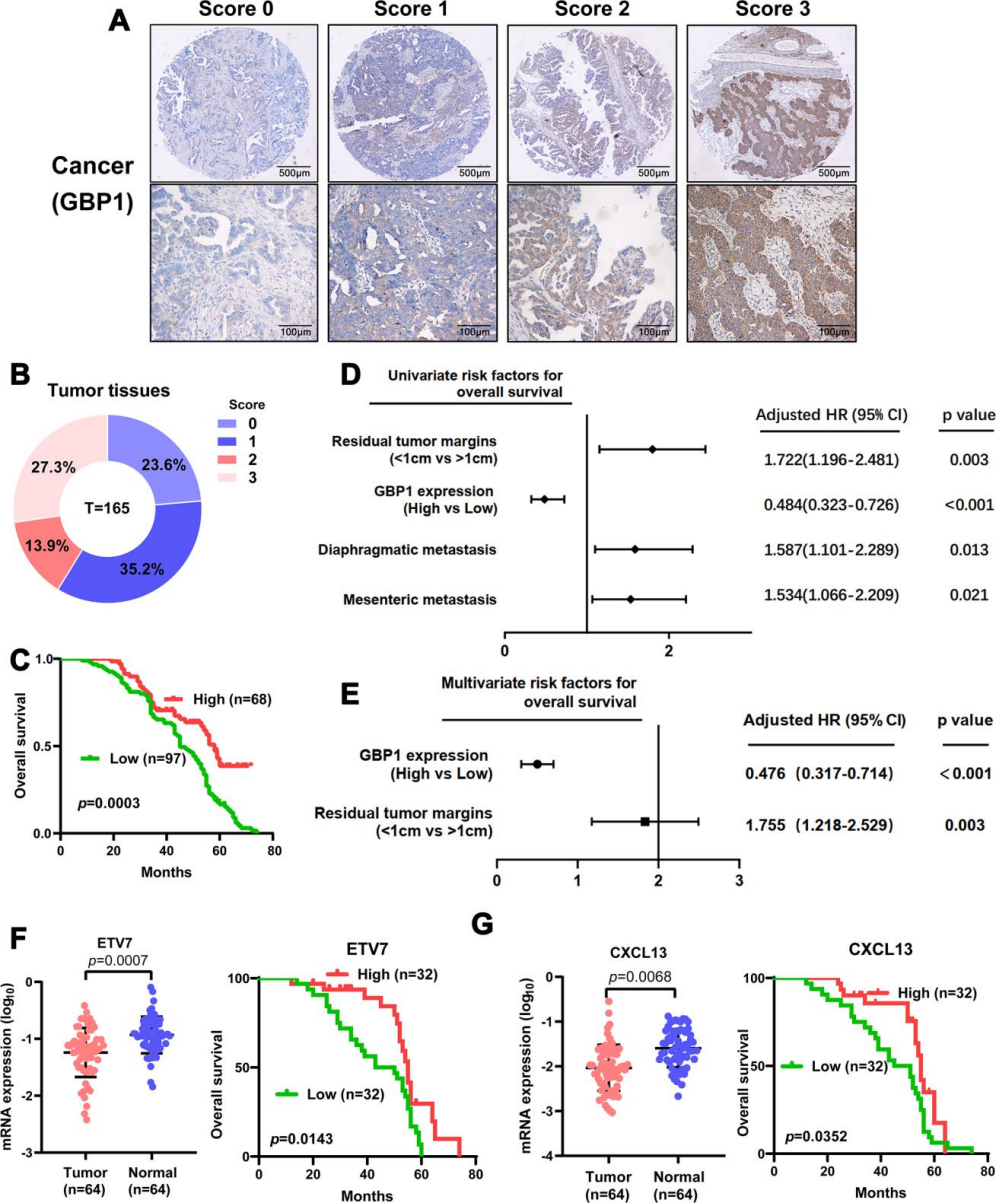
**Levels of GBP1 and ETV7 expression correlated with overall survival in HGSOC.** (**A**) Representative images of GBP1 expression in HGSOC tissues, visualized at 40× and 200× magnification. (**B**) Distribution of the immunoreactive score (IRS) in an HGSOC TMA. (**C**) Kaplan-Meier survival curve with log-rank analysis of overall survival showed statistical significance between the curves of patients with high GBP1 expression and those with low GBP1 expression (log-rank test, *p*=0.0003). (**D**) Univariate analysis was performed in 165 HGSOC patients. All bars correspond to 95% confidence intervals. (**E**) Multivariate analysis was performed in 165 HGSOC patients. All bars correspond to 95% confidence intervals. (**F–G**) Expression levels of ETV7 and CXCL13 were measured in HGSOC tissues compared with controls. Moreover, Kaplan-Meier method indicated the prognostic significance of ETV7 and CXCL13.

To further validate the clinical importance of GBP1, ETV7 and CXCL13, we aimed to perform immunostaining with antibodies on microarrays containing tissue from 165 HGSOC patients. Given that no antibodies specific for ETV7 and CXCL13 are commercially available, RT-PCR was adopted to confirm their expression levels and prognostic significance. We first assessed the protein expression of GBP1. Positive immunostaining signals of GBP1 were seen in the cytoplasm and membrane (Figure 7A). Of 165 patients with HGSOC, low expression (score 0-1) was observed in 94, and high expression (score 2-3) was observed in 71 (Figure 7B). The protein level of GBP1 was not correlated with any pathological characteristics of HGSOC but was potentially correlated with diaphragmatic metastasis (*p*=0.05; Table 2). We then plotted the overall survival curves according to GBP1 protein levels using the Kaplan-Meier method. As shown in Figure 7C, patients with high GBP1 protein expression exhibited more favorable overall survival (*p*=0.0003). Univariate analysis of overall survival revealed that GBP1 levels (*p* < 0.001), the residual tumor margins (*p*=0.003), diaphragmatic metastasis (p = 0.013) and mesenteric metastasis (*p*=0.021) were prognostic indicators in HGSOC (Figure 7D). Further multivariate analysis revealed that GBP1 expression (*p* < 0.001) and the residual tumor margins (*p*=0.003) were independent predictors for overall survival in HGSOC patients (Figure 7E). Moreover, we observed decreased ETV7 and CXCL13 mRNA expression in HGSOC tissues compared with normal ovary tissues (Figure 7F–7G). Kaplan-Meier analysis indicated that ETV7 and CXCL13 mRNA expression correlated with positive OS (*p*<0.05, Figure 7F–7G). Of note, the prognostic results of CXCL13 contradicted our *in vitro* experiments, perhaps because mRNA expression is not always consistent with protein levels. In addition, CXCL13, which is a cytokine, may exert its functions outside cells. In general, we demonstrated the prognostic value of GBP1 and ETV7 in HGSOC patients. Besides that, CXCL13 may require further large samples validation.

**Table 2:** Correlation between GBP1 and clinicopathological parameters.

**Variable**	**n (%)**	**Expression of GBP1**		**χ^2^**	***p*-value**
		**Low**	**High**		
Age (years)				1.311	0.252
≤ 65	140 (84.8)	78	62		
> 65	25 (15.2)	17	8		
Size (cm)				0.057	0.811
≤ 5	89 (53.9)	52	37		
> 5	76 (46.1)	43	33		
FIGO stage				0.198	0.657
III	157 (95.2)	91	66		
IV	8 (4.8)	4	4		
Residual tumor margins				3.177	0.210
≤1 cm	113 (68.5)	59	54		
>1 cm	52 (31.5)	35	17		
Ascites (ml)			0.455	0.500
≤1500	94 (57.0)	52	42		
>1500	71 (43.0)	43	28		
Diaphragmatic metastasis			3.830	0.050
Yes	83 (50.3)	54	29		
No	82 (49.7)	41	41		
Mesenteric metastasis			2.991	0.084
Yes	86 (52.1)	55	31		
No	79 (47.9)	40	39		

## DISCUSSION

Despite advances in surgery and targeted therapy for ovarian cancer, 70% of women still succumb to this disease. The identification of effective prognostic biomarkers for HGSOC could enhance clinical decision making. Recently, immune profiling in cancers, which is based on immune cell distribution and density, has become an important indicator for prognostic evaluations. In this study, we used the CIBERSORT algorithm and, for the first time, combined the calculated TMEscore with HGSOC characteristics to explore TME-related prognostic genes.

Numerous studies to date have developed immune-relevant signatures to stratify cancer survival, detect recurrence, and distinguish benign from malignant masses based on different cohorts [19, 20]. For example, D Zeng [10] constructed an immunoscore to estimate OS in patients with gastric cancer. XB Pan [8] provided a more comprehensive understanding of the TME as well as a list of prognostic immune-related genes in cervical cancer by public database analysis. L Deng [7] suggested that plasma cells and CD4^+^ central memory T (Tcm) cells in the TME may play a role in the subsequent progression of triple-negative breast cancer. WH Xu [9] obtained a list of TME-related genes with prognostic value using immune/stromal scores after processing by the ESTIMATE algorithm in multiple cohorts. However, these studies focused only on bioinformatic analyses. To our knowledge, ours is a novel study incorporating the TCGA database and experimental exploration to confirm the prognostic significance of the TMEscore in HGSOC.

To explore the potential mechanisms underlying changes in the TME in HGSOC, we derived TME-related genes for further use in functional enrichment analysis and PPI network construction. We identified a total of 329 DEGs by comparing the high vs. low TMEscore groups, many of which were involved in immune pathways, such as T cell activation, CXCR chemokine receptor binding and chemokine activity. This finding is consistent with those of previous intrinsic studies showing that the functions of immune cells and chemokines are interrelated in the establishment of the TME in HGSOC. Further analysis via additional validation in public datasets revealed seven significant genes (GBP1, ETV7, CXCL13, UBD, TAP1, GBP5, and PLA2G2D) related to HGSOC outcomes. Guanylate-Binding Protein-1 (GBP1) is a member of the large GTPase family and is induced by interferons and inflammatory cytokines [21–23]. ETV7 (also referred to as TEL2) is a member of the ETS transcription factor family and plays a key role in hematopoiesis [24–26]. Chemokine (C-X-C motif) ligand 13 (CXCL13) is a B cell-attracting chemokine that serves as an important factor during tumor proliferation and migration [27, 28]. UBD, a member of the ubiquitin-like protein (UBL) family, is involved in various essential cellular development processes, including immune-mediated inflammation, apoptosis, cell cycle progression, and proliferation [29, 30]. Transporter associated with antigen processing 1 (TAP1) is essential for peptide delivery from the cytosol to the lumen of the endoplasmic reticulum in the major histocompatibility complex (MHC) class I antigen-presenting pathway [31]. As described above, Guanylate-binding protein (GBP) 5 belongs to the GBP family, which is involved in important cellular processes, including signal transduction, translation, vesicle trafficking, and exocytosis [32]. The PLA2G2D protein is expressed in human monocyte-derived macrophages, nasal epithelial cells and bronchial epithelial cells following inflammatory stimulation with, for example, interferon-γ [33].

Given that the TME plays a critical role in the progression of tumors, we also preliminarily investigated the role of TME-derived genes *in vitro*. We were particularly interested in the three genes with the lowest *p*-values in the prognostic analysis (GBP1, ETV7 and CXCL13). Cellular verification demonstrated that GBP1 and ETV7 may act as tumor suppressors in HGSOC, whereas CXCL13 may promote cell proliferation and migration. These findings are consistent with those of previous studies, although the functions of GBP1 and ETV7 in different tumors are controversial [21, 22, 34, 35]. Moreover, we conducted immunohistochemistry (IHC) experiments to assess the expression of GBP1. Multivariate analysis revealed that GBP1 expression was an independent prognostic predictor in HGSOC patients. Notably, CXCL13 was specifically associated with more favorable OS, a finding contradicted by our experimental results. Ignacio RMC arrived at the same conclusion regarding survival in p53 mutant serous OC [36]. The specific reason may be attributed to the ascites of HGSOC and our use of RT-PCR, but further studies are needed to confirm these findings.

Our study suffered from some limitations. First, the TME-related prognostic genes were identified from the TCGA database. These genes should be further validated in large prospective clinical trials. Second, the methodology for interpreting immune infiltration and the appropriate cutoff value needs to be standardized. Third, considering tumor heterogeneity, single-cell sequencing may more accurately reflect changes in the TME. Fourth, more mechanistic studies are required to further elucidate the biological functions underlying the discovered prognostic genes. Nonetheless, we identified several prognostic genes related to the TME that may become new effective prognostic biomarkers for HGSOC.

## MATERIALS AND METHODS

### Ethics statement

The patients samples were used with approval from the Ethics Committee of Fudan University Shanghai Cancer Center and with consent from all patients. All procedures were performed in accordance with the Declaration of Helsinki and relevant policies in China.

### Public data processing and TMEscore calculation

Level 3 data of HGSOC patients with available clinicopathological and survival data were downloaded from the TCGA data coordination center (https://tcga-data.nci.nih.gov/tcga/). For validation, gene expression profiles and clinical survival data for OC patients were obtained from the ICGC data portal (Supplementary Table 7, https://icgc.org/).

Based on the expression profile and clinical data downloaded from TCGA, heteroscedasticity was removed using the edgeR voom algorithm, and the proportion of LM22 cells was calculated with CIBERSORT, which allows sensitive and specific discrimination of 22 human immune cell phenotypes, including B cells, T cells, natural killer cells, macrophages, dendritic cells (DCs), and myeloid subsets [14, 15]. Then, according to the obtained cell proportions, three unsupervised clustering methods were used to explore sample classification, and the differences in expression were analyzed. According to the differential gene expression values obtained, consistency clustering was conducted to explore sample classification, and a chi-square test of independence was used to determine whether the two classifications before and after the three methods were consistent. Via a random forest model, signature genes were obtained by removing the redundancy of differentially-expressed genes (DEGs) with consistent classification. A Cox regression model was used to detect the relationship between signature genes and sample survival and to classify the genes. Finally, the TMEscores associated with every sample were calculated according to the gene classification. Data processing and TMEscore construction methods are detailed in the Supplementary Methods.

### DEGs associated with the TMEscore

DEG analysis was performed with the Limma package [37] according to the median TMEscore value. The samples were separated into high and a low TMEscore groups. An empirical Bayesian approach was applied to estimate the gene expression changes using moderated *t* tests. The adjusted *p*-values for multiple testing were calculated using the Benjamini-Hochberg correction. A |log(fold change)|≥1 and an adjusted *p*-value < 0.05 were set as the cutoff criteria to screen for DEGs.

### Module analysis and PPI network construction

The PPI network was retrieved from the STRING database [38]. Molecular interaction networks were visualized using Cytoscape software. Only individual networks with 10 or more nodes were included in further analyses. The connectivity degree of each node in the network was calculated. MCODE was then used to find clusters based on topology to locate densely connected regions [39].

### Pathway enrichment analysis for molecular function

To further understand the functions of prognosis-related genes, we performed pathway enrichment analysis with the genes based on the KEGG and GO databases, including biological process (BP), molecular function (MF) and cellular component (CC) categories [40]. The top-ranked pathways with an adjusted *p*-value < 0.05 were identified as statistically significant. All analyses were performed using the R package ClusterProfiler [41].

### Survival analysis

Kaplan-Meier plots were generated to illustrate the relationship between patient overall survival and the levels of DEGs. DEGs were identified as binary variables (high vs. low) using the median expression value as the cutoff value. A log-rank test was used to assess differences between survival curves.

### Cell culture and treatments

The human OC cell line A2780 was cultured in Dulbecco’s modified Eagle’s medium (Gibco, Carlsbad, CA, USA) supplemented with 10% fetal bovine serum (FBS) (Gibco), 50 U/mL of penicillin and 50 μg/mL of streptomycin (Gibco). A2780 cell line was maintained at 37 °C and 5% CO2 in a humidified atmosphere.

### Cell transfections and western blot analysis

SiRNAs targeting GBP1, ETV7, CXCL13 were purchased from GenePharma (Shanghai, China). Lipofectamine 3000 (Invitrogen, USA) was used for transfection experiments. All siRNA sequences are listed in Supplementary Table 8.

Transfected cells were cultured for 60 h and were then lysed in RIPA buffer (Sigma-Aldrich, USA) supplemented with a phosphatase inhibitor (Roche) and a protease inhibitor (Roche, Basel, Switzerland). A BCA protein assay kit (Thermo Scientific, USA) was used to measure protein concentration. The collected cell lysates were separated on 10% sodium dodecyl sulfate-polyacrylamide gel electrophoresis (SDS-PAGE) gels (EpiZyme, Shanghai, China) and were then transferred to polyvinylidene fluoride (PVDF) membranes (Millipore, Billerica, MA), which were blocked with 5% milk for 1 h at room temperature. PVDF membranes were incubated with primary antibodies (anti-β-actin, anti-GBP1, anti-ETV7, anti-CXCL13 antibodies, purchased from Proteintech) overnight at 4 °C, washed, and incubated with a secondary antibody for 1 h at room temperature. Signals were detected using chemiluminescence. β-Actin was used as the endogenous loading control.

### Cell proliferation assays

A total of 2×10^3^ cells per well were seeded in 96-well plates 24 h before the experiment. A2780 cells were transfected with the targeting siRNAs or scrambled siRNA. Proliferation was measured using a CCK-8 kit (BOSTER, China) according to the manufacturer’s protocol. All experiments were performed in triplicate. Cell proliferation curves were plotted using the absorbance at each time point.

Transfected cells were digested with trypsin into single-cell suspensions 48 h later. For the colony formation assay, a total of 500 or 1,000 cells were plated in 12-well plates and incubated in corresponding medium containing 10% FBS at 37 °C. After being cultured for two weeks, the cells were fixed with anhydrous alcohol for 30 min and stained with 0.1% crystal violet for approximately 15 min. Then, visible colonies were manually counted. Triplicate wells were analyzed for each treatment group.

### Migration assays

A cell invasion assay was performed using Transwell chamber inserts (8.0 mm, Corning, NY, USA) in a 24-well plate. Then, 4×10^4^ cells suspended in 200 μL of serum-free medium were added to the upper chambers. Culture medium containing 20% FBS was placed in the bottom chambers. The cells were incubated for 36 or 48 h at 37 °C. After incubation, the cells on the upper surface were scraped and washed away, whereas the cells on the lower surface were fixed with 20% methanol and stained with 0.1% crystal violet. The numbers of invaded cells in five randomly selected fields were counted using a microscope. The experiments were repeated independently in triplicate.

### Immunohistochemistry

Immunohistochemistry was performed using the Envision System with diaminobenzidine (Gene Tech Co., Ltd., Shanghai) according to the manufacturer’s protocol. HGSOC tissue microarrays (TMAs) were constructed by Shanghai Outdo Biotech. In brief, specimens were incubated first with an anti-GBP1 antibody (1:200, Proteintech, China) overnight at 4 °C and then with a biotinylated secondary antibody (1:100, goat anti-rabbit IgG) for 30 min at 37 °C.

### RNA isolation, reverse transcription and quantitative real-time PCR

Total RNA was extracted from the tissue samples using TRIzol reagent (Invitrogen, Carlsbad, CA, USA) according to the manufacturer’s protocol. Reverse transcription (RT) and quantitative real-time PCR (qRT-PCR) kits (Takara, Dalian, China) were utilized to evaluate the mRNA levels of the indicated genes. PCR primers were designed and synthesized using a primer design tool (Vector NTI®); The primers used in this study were listed as follows: ETV7: 5′-CTG CTG TGG GAT TAC GTG TAT C-3′ (Forward) and 5′-GTT CTT GTG ATT TCC CCA GAG TC-3′ (Reverse); CXCL13: 5′-GCT TGA GGT GTA GAT GTG TCC-3′ (Forward) and 5′-CCC ACG GGG CAA GAT TTG AA-3′ (Reverse). The relative quantification value for each target gene was expressed as 2^−ΔΔCT^. β-Actin was used as an internal reference.

### Statistical analysis

RStudio software (Version 1.2.1335) and GraphPad Prism 8 were used to construct the plots shown in the figures. Statistical analyses were conducted using RStudio software (Version 1.2.1335) and SPSS 22.0 (SPSS, Inc., Chicago, IL, USA). A value of *P*<0.05 indicated a statistically significant difference.

## Supplementary Material

Supplementary Method

Supplementary Figure 1

Supplementary Table 1

Supplementary Table 2

Supplementary Table 3

Supplementary Table 4

Supplementary Table 5

Supplementary Table 6

Supplementary Table 7

Supplementary Table 8
