# Supplementary material for: Immune profiling reveals prognostic genes in high-grade serous ovarian cancer
Source: Aging (Albany NY). 2020 Jun 16;12(12):11398–415. doi: 10.18632/aging.103199 (PMC7343445; doi:10.18632/aging.103199)
Supplement: Supplementary Method [file aging-12-103199-s005..pdf]

SUPPLEMENTARY METHOD

TMEscore construction

To quantify the proportions of immune cells in the ovarian cancer samples, we used the CIBERSORT algorithm and the LM22 gene signature, which allows for highly sensitive and specific discrimination of 22 human immune cell phenotypes, including B cells, T cells, natural killer cells, macrophages, DCs, and myeloid subsets. CIBERSORT is a deconvolution algorithm that uses a set of reference gene expression values (a signature with 547 genes) considered a minimal representation for each cell type and, based on those values, infers cell type proportions in data from bulk tumor samples with mixed cell types using support vector regression. Gene expression profiles were prepared using standard annotation files, and data were uploaded to the CIBERSORT web portal (http://cibersort.stanford.edu/), with the algorithm run using the LM22 signature and 1,000 permutations. Tumors with qualitatively different TME cell infiltration patterns were grouped using hierarchical agglomerative clustering (based on Euclidean distance and Ward's linkage). Unsupervised clustering methods (K-means) for dataset analysis were used to identify TME patterns and classify patients for further analysis. Principal component analysis (PCA) was conducted. Principal component 1 was extracted to serve as the gene signature score. After obtaining the prognostic value of each gene signature score, we applied a method similar to GGI to define the TMEscore of each patient: TMEscore = $\sum$ PC1i – $\sum$PC1j where i is the signature score whose Cox coefficient is positive, and j is the expression level of genes whose Cox coefficient is negative.
