# Supplementary material for: Immune profiling reveals prognostic genes in high-grade serous ovarian cancer
Source: Aging (Albany NY). 2020 Jun 16;12(12):11398–415. doi: 10.18632/aging.103199 (PMC7343445; doi:10.18632/aging.103199)
Supplement: Supplementary Figure 1 [file aging-12-103199-s007..pdf]

SUPPLEMENTARY FIGURE

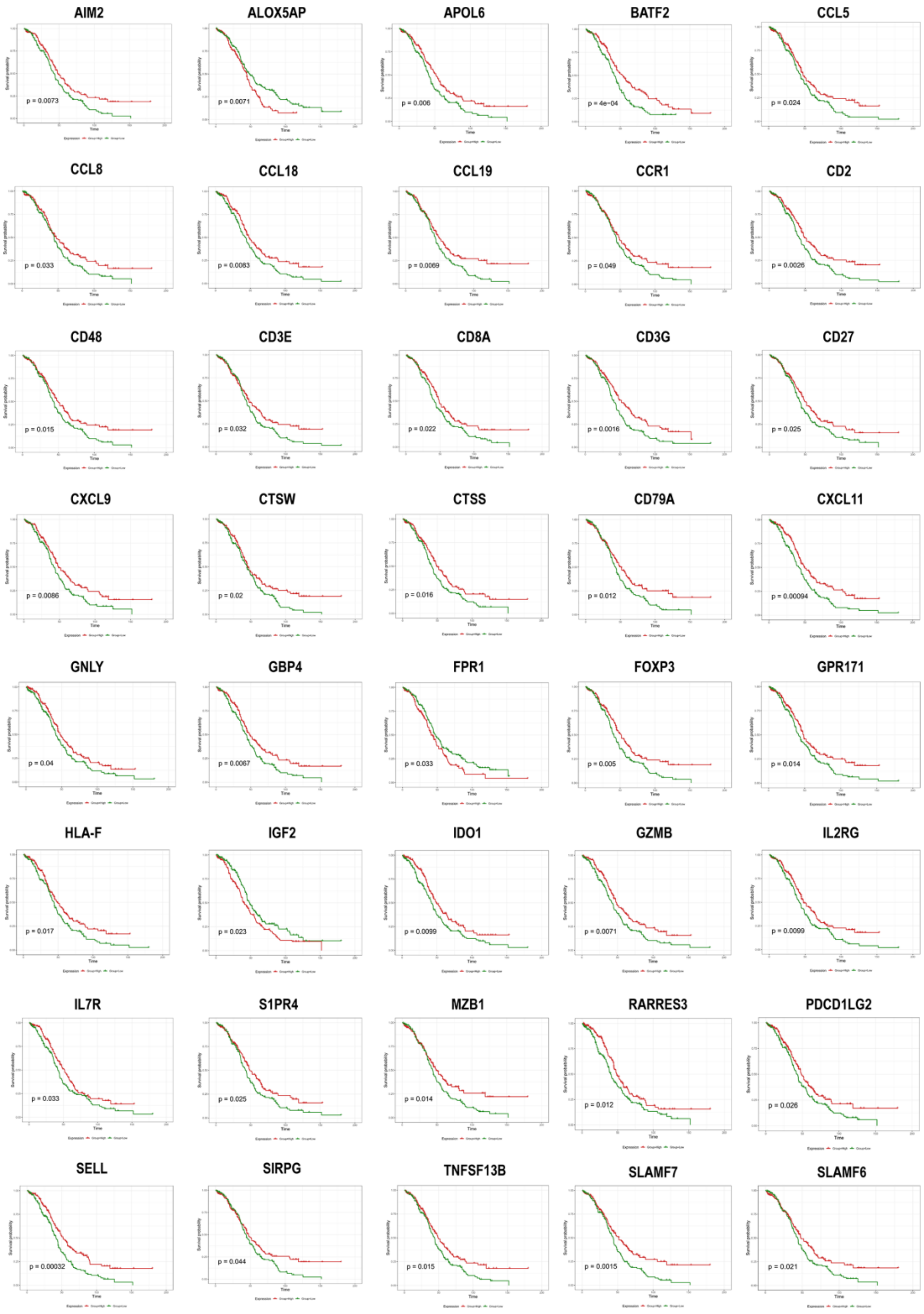

Supplementary Figure 1. The survival analysis of DEGs in HGSOC. OS=overall survival time in months.
