# Supplementary material for: Immune profiling reveals prognostic genes in high-grade serous ovarian cancer
Source: Aging (Albany NY). 2020 Jun 16;12(12):11398–415. doi: 10.18632/aging.103199 (PMC7343445; doi:10.18632/aging.103199)
Supplement: Supplementary Table 8 [file aging-12-103199-s006..pdf]

Supplementary Table 8. Oligonucleotide sequences of siRNAs.

Name	Sequence (5' - 3')
aGBP1 (human) siRNA-1	GAU ACA GGC UGA AGA GAU UTT
GBP1 (human) siRNA-2	GCA CAG GGA CAG UGA GAG ATT
GBP1 (human) siRNA-3	GAA CAG AAG GAG AGG AGU UTT
ETV7 (human) siRNA-1	GGG AAG ACA AGG ACG CCA ATT
ETV7 (human) siRNA-2	ACA AGA ACC GGG UGA ACA UTT
ETV7 (human) siRNA-3	GCU GUG GGA UUA CGU GUA UTT
CXCL13 (human) siRNA-1	UGA UGG AAG UAU UGA GAA ATT
CXCL13 (human) siRNA-2	GGA AGA AGA ACA AGU CAA UTT
CXCL13 (human) siRNA-3	GGG AAU GGU UGU CCA AGA ATT
